# The genome sequence of black currant, *Ribes nigrum* L. (Saxifragales: Grossulariaceae)

**DOI:** 10.12688/wellcomeopenres.28122.1

**Published:** 2026-10-13

**Authors:** Maarten J. M. Christenhusz, Ilia J. Leitch, Michael F. Fay, Royal Botanic Gardens Kew Genome Acquisition Lab, Plant Genome Sizing Collective, Darwin Tree of Life Barcoding collective, Wellcome Sanger Institute Tree of Life Management, Samples and Laboratory team, Wellcome Sanger Institute Scientific Operations: Sequencing Operations, Wellcome Sanger Institute Tree of Life Core Informatics team, Tree of Life Core Informatics collective, Darwin Tree of Life Consortium

**Affiliations:** 1 Royal Botanic Gardens Kew, Richmond, Surrey, England, UK; 2 Botanical Society of Britain and Ireland, St Albans, Hertfordshire, England, UK

**Keywords:** Ribes nigrum, black currant, genome sequence, chromosomal, Saxifragales

## Abstract

We present a genome assembly of *Ribes nigrum* (black currant; Streptophyta; Magnoliopsida; Saxifragales; Grossulariaceae). The assembly consists of two haplotypes with total lengths of 894.28 megabases and 883.57 megabases. Most of the haplotype 1 assembly (96.1%) was scaffolded into eight chromosomal pseudomolecules. Haplotype 2 was assembled to scaffold level. The mitochondrial genome assembly has a length of 450.22 kilobases and the plastid genome assembly has a length of 157.45 kilobases. This assembly was generated as part of the Darwin Tree of Life project, which produces reference genomes for eukaryotic species found in Britain and Ireland.

## Species taxonomy

Eukaryota; Viridiplantae; Streptophyta; Streptophytina; Embryophyta; Tracheophyta; Euphyllophyta; Spermatophyta; Magnoliopsida; Mesangiospermae; eudicotyledons; Gunneridae; Pentapetalae; Saxifragales; Grossulariaceae; *Ribes*; *Ribes nigrum* L. (NCBI:txid78511)

## Background

As part of the Darwin Tree of Life Project, a collaborative effort to sequence all named eukaryotic species in the Atlantic Archipelago of Britain and Ireland, we sequenced the genome of black currant, *Ribes nigrum* L.

*Ribes nigrum* is a spineless, deciduous shrub growing to 1.5 m (occasionally 2 m) tall. Leaves are alternate and three- to five-lobed and smell strongly of currants due to the presence of scattered glands on the underside of the leaf. The flowers are minute with five pale green petals and are frequently visited by bees, although this species is predominantly self-pollinating. The fruit, which is an edible berry containing several seeds, turns dark purple to black when ripe ( Sell & Murrell, 2014).

*Ribes nigrum* has a wide native distribution from France to northeast Asia. Beyond this, it is widely naturalised in countries to the south of its native range as well as in Britain, Ireland and eastern North America ( POWO, 2026). It is normally considered a neophyte in Britain and Ireland as it was first recorded growing in cultivation in the early seventeenth century following its introduction from The Netherlands by Trandescant ( Hummer & Barney, 2002). The first reports of growing wild in Kent and Bedfordshire were not until 1657 and 1660 ( Preston, 2018). Nevertheless, some localised populations in fen-carr and wet woodlands in East Anglia have been suggested to be native ( Croft & Walker, 2023). It is now widely distributed as an escape from cultivation in the south of Britain, becoming less common in northern Scotland and in parts of Ireland.

Black currant is an alternate host for the fungus *Cronartium ribicola* J.C.Fisch. which causes white pine blister rust, a disease that kills some North American five-needled pines. As a result of the risk to timber production, growing black currant was forbidden in the USA for much of the 20 ^th^ century. Nevertheless, cultivars of *R. nigrum* have now been developed that do not carry the disease, and there is now renewed interest in black currants as a crop in the USA ( Cortez & Gonzalez de Mejia, 2019).

The main use of black currant is in the production of foodstuffs including preserves and cordials from the fruits. They normally contain added sugar due to the bitter and astringent flavour of the fruits. The fruits contain high concentrations of vitamin C and flavonoids including anthocyanins, with reported antioxidant, antimicrobial and anti-inflammatory properties ( Cortez & Gonzalez de Mejia, 2019). As a result, they have been widely used in traditional medicine, e.g. as a hot drink for soothing sore throats.

We present a chromosomally complete genome sequence of *Ribes nigrum* (Figure 1) based on a specimen collected from Wolsey Drive Allotments, Kingston upon Thames, Surrey, United Kingdom. Most of the assembly is scaffolded into eight chromosomal pseudomolecules, reflecting the gametophytic (1 *n*) chromosome count of this species which is reported to be a diploid with a sporophytic chromosome number of 2 *n* = 2 *x* = 16 ( Machlańska *et al.*, 2026; Mohammadpour *et al.*, 2023). This genome will provide a valuable genomic resource for this species, including insights into the bioactive compounds produced in black currant and related species and the resilience of this crop species in the face of climate change.

Another chromosome-level assembly for this species is available (GCA_965236665.2; submitted by Heinricht Heine Universitaet Duesseldorf).

**Figure 1.**
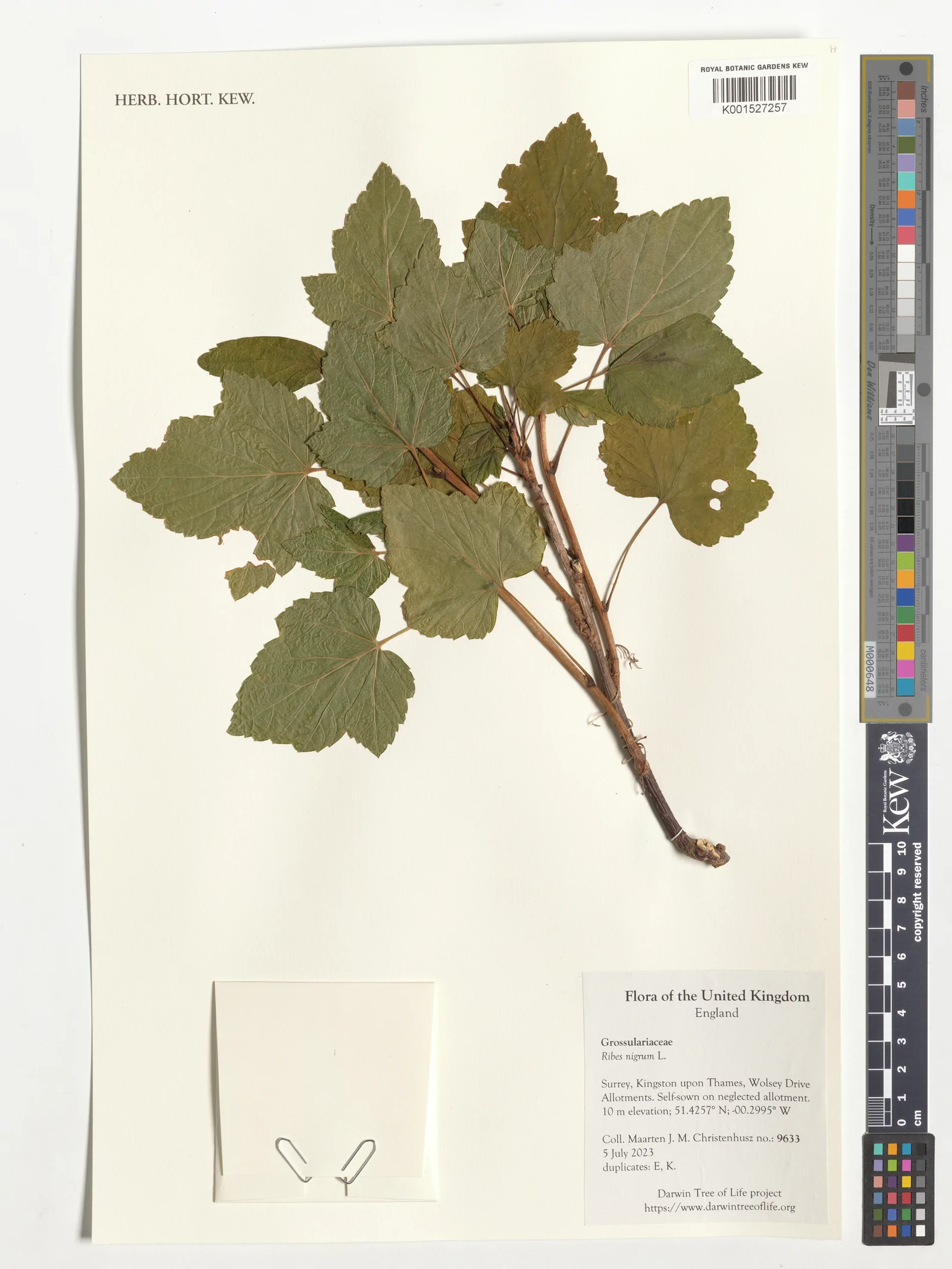
Herbarium voucher of the sequenced *Ribes nigrum* plant (drRibNigr1), Kew K001527257.

## Methods

### Sample acquisition, flow cytometry and DNA barcoding

A specimen of *R. nigrum* (specimen ID KDTOL10979, ToLID drRibNigr1) was used for genome sequencing. It was collected from Kingston upon Thames, Surrey, England, UK (latitude 51.4257, longitude −0.2995) on 2023-07-05. The specimen was collected and identified by Maarten Christenhusz. The drRibNigr1 specimen was also used for Hi-C and RNA sequencing. The herbarium voucher for the sequenced plant (collector number MC9633) is deposited at Kew ( K001527257); the voucher is shown in Figure 1.

The genome size was estimated by flow cytometry following the ‘one-step’ method outlined in Pellicer *et al*. (2021) and using propidium iodide as the fluorochrome. The General Purpose Buffer (GPB) supplemented with 3% PVP was used for isolation of nuclei ( Loureiro *et al.*, 2007), and the internal calibration standard was *Petroselinum crispum* ‘Champion Moss Curled’ with an assumed 1C-value of 2,200 Mb ( Obermayer *et al.*, 2002).

The initial identification was verified by an additional DNA barcoding process according to the framework developed by Twyford *et al*. (2024). Part of the plant specimen was preserved in silica gel desiccant ( Chase & Hills, 1991). DNA extracted from the dried plant was amplified by PCR for standard barcode markers, with the amplicons sequenced and compared to public sequence databases including GenBank and the Barcode of Life Database (BOLD) ( Ratnasingham & Hebert, 2007). Following whole-genome sequencing, the relevant DNA barcode region was also used alongside the initial barcoding data for sample tracking at the WSI ( Twyford *et al.* , 2024). The standard operating procedures for Darwin Tree of Life barcoding are available on protocols.io.

### Nucleic acid extraction

High molecular weight (HMW) DNA extraction protocols developed by the Tree of Life Core Laboratory at the Wellcome Sanger Institute (WSI) are available on protocols.io ( Howard *et al.*, 2025). Tissue from the drRibNigr1 sample was weighed and triaged to select the appropriate extraction workflow.

For library DTOL14819228, 87.0 mg of leaf tissue was homogenised by cryogenic bead beating. HMW DNA was extracted using the Plant MagAttract v4 protocol. DNA was fragmented following the Diagenode Megaruptor3 for LI PacBio protocol, and purified following the Sanger Tree of Life Fragmented DNA clean up: Automated SPRI protocol.

The concentration of the sheared and purified DNA was assessed with a NanoDrop spectrophotometer and a Qubit Fluorometer using the Qubit dsDNA High Sensitivity Assay kit. Fragment size distribution was evaluated by running the sample on the Femto Pulse system. For this sample, final post-shearing DNA measurements included a Qubit concentration of 28 ng/μL, an average fragment size of 15.3 kb and a yield of 1,540.00 ng. The Genomic Quality Number (GQN) was 6.7.

RNA was extracted from leaf tissue of drRibNigr1 in the Tree of Life Laboratory at the WSI using the RNA Extraction: Automated MagMax™ *mir*Vana protocol. The RNA concentration was assessed with a NanoDrop spectrophotometer and a Qubit Fluorometer using the Qubit RNA Broad-Range Assay kit. RNA integrity was assessed using the Agilent RNA 6000 Pico Kit and Eukaryotic Total RNA assay.

### PacBio HiFi library preparation and sequencing

Library DTOL14819228 was prepared with the SMRTbell Prep Kit 3.0 (Pacific Biosciences) according to the manufacturer’s instructions. The kit includes reagents for end repair/A-tailing, adapter ligation, post-ligation SMRTbell bead clean-up, and nuclease treatment. Size selection and clean-up were performed using diluted AMPure PB beads (Pacific Biosciences). DNA concentration was quantified with a Qubit Fluorometer v4.0 (Thermo Fisher Scientific) and the Qubit 1X dsDNA HS assay kit. Final library fragment size was assessed with the Agilent Femto Pulse Automated Pulsed Field CE Instrument (Agilent Technologies) using the gDNA 55 kb BAC analysis kit.

The library was sequenced on a Revio instrument (Pacific Biosciences, California, USA). Prepared libraries with a molarity greater than 2 nM were normalised to 2 nM, and 15 μL was used for making complexes. For libraries with a molarity below 2 nM, the available 10 μL library volume was used without normalisation. Primers were annealed and polymerases were bound to generate circularised complexes, following the manufacturer’s instructions. Complexes were purified using 1.2× SMRTbell beads, diluted to the Revio loading concentration of 200–300 pM, and spiked with a Revio sequencing internal control. Sequencing used Revio 25M SMRT cells. SMRT Link software (Pacific Biosciences), a web-based workflow manager, was used to configure and monitor the run and to carry out primary and secondary data analysis.

### Hi-C

***Sample preparation and crosslinking***

Hi-C data were generated from the drRibNigr1 leaf sample using the Arima-HiC v2 kit (Arima Genomics). Tissue was finely ground using the Covaris cryoPREP Dry Pulverizer (Covaris), and then subjected to nuclei isolation. Nuclei were isolated using a modified protocol based on the Qiagen QProteome Cell Compartment Kit (Qiagen), in which only the Lysis and CE2 buffers were used, with QIAshredder spin columns. After isolation, nuclei were fixed using formaldehyde to a final concentration of 2% to crosslink the DNA. The crosslinked DNA was then digested and biotinylated according to the manufacturer’s instructions. A clean-up step was performed with SPRIselect beads before library preparation. DNA concentration was quantified using the Qubit Fluorometer v4.0 (Thermo Fisher Scientific) and the Qubit HS Assay Kit, following the manufacturer’s instructions.

***Hi-C library preparation and sequencing***

Biotinylated DNA constructs were fragmented using a Covaris E220 sonicator and size-selected to 400–600 bp using SPRISelect beads. DNA was enriched with Arima-HiC v2 kit Enrichment beads. End repair, A-tailing, and adapter ligation were carried out with the NEBNext Ultra II DNA Library Prep Kit (New England Biolabs), following a modified protocol where library preparation occurs while DNA remains bound to the Enrichment beads. Libraries were amplified with KAPA HiFi HotStart mix and a custom Unique Dual Index (UDI) barcode set (Integrated DNA Technologies). Depending on sample concentration and biotinylation percentage determined at the crosslinking stage, libraries were amplified with 10–16 PCR cycles. Post-PCR clean-up was performed with SPRISelect beads. Libraries were quantified using the AccuClear Ultra High Sensitivity dsDNA Standards Assay Kit (Biotium) and a FLUOstar Omega plate reader (BMG Labtech).

Prior to sequencing, libraries were normalised to 10 ng/μL. Normalised libraries were quantified again to create equimolar and/or weighted 2.8 nM pools. Pool concentrations were checked using the Agilent 4200 TapeStation (Agilent) with High Sensitivity D500 reagents before sequencing. Libraries were sequenced on the Illumina NovaSeq X, generating paired-end 150-bp reads.

### RNA library preparation and sequencing

Libraries were prepared with the NEBNext® Ultra II Directional RNA Library Prep Kit for Illumina (New England Biolabs), following the manufacturer’s instructions. Poly(A) mRNA was isolated from 100 ng of total RNA using oligo(dT) beads, converted to cDNA, and uniquely indexed; 14 PCR cycles were performed. Libraries were prepared with an intended average fragment size of 100–300 bp. Libraries were quantified, normalised, and pooled equimolarly to a final concentration of 2.8 nM before dilution to 150 pM for loading. Sequencing was carried out on the Illumina NovaSeq X, generating paired-end reads.

### Genome assembly

Prior to assembly of the PacBio HiFi reads, a database of *k*-mer counts ( *k* = 31) was generated from the filtered reads using FastK. The *k*-mer frequency distribution was analysed with GenomeScope (version 2.1.0) ( Ranallo-Benavidez *et al.*, 2020), providing fitted point estimates of haploid genome size, heterozygosity and repeat content.

The HiFi reads were assembled using Hifiasm in Hi-C phasing mode ( Cheng *et al.*, 2021, 2022), producing two haplotypes. Hi-C reads ( Rao *et al.* , 2014) were mapped to contigs using bwa-mem2 ( Vasimuddin *et al.*, 2019). Contigs were further scaffolded with Hi-C data in YaHS ( Zhou *et al.*, 2023), using the --break option for handling potential misassemblies. The scaffolded assemblies were evaluated using Gfastats ( Formenti *et al.* , 2022), BUSCO ( Manni *et al.*, 2021; Tegenfeldt *et al.*, 2025) and MerquryFK ( Rhie *et al.*, 2020). The organelle genomes were assembled using OATK ( Zhou *et al.*, 2025). The comparator for the mitochondrial assembly was the mitochondrial genome of *Ribes nigrum* (NC_082306.1). The comparator for the plastid assembly was the chloroplast genome of *Ribes nigrum* (OR227936.1).

### Assembly curation

The assembly was decontaminated using the Assembly Screen for Cobionts and Contaminants ( ASCC) pipeline. The flat files and maps for curation were generated with TreeVal. Manual curation was conducted primarily in PretextView and HiGlass ( Kerpedjiev *et al.* , 2018). Scaffolds were visually inspected and corrected as described by Howe *et al*. (2021). The two haplotype assemblies were combined for curation. Manual corrections included 27 breaks and 26 joins. The curation process is described at sanger-tol/curation-resources. A Hi-C contact map of the haplotype 1 assembly was generated with PretextSnapshot.

### Assembly quality assessment

The MerquryFK tool ( Rhie *et al.*, 2020) was run in a Singularity container ( Kurtzer *et al.*, 2017) to evaluate *k*-mer completeness and assembly quality for both haplotypes using the *k*-mer database ( *k* = 31) computed prior to genome assembly. The analysis outputs included assembly QV scores and completeness statistics.

The haplotype 1 assembly was assessed using BlobToolKit ( Challis *et al.*, 2020). PacBio reads were mapped to the assembly to derive sequence coverage, and BUSCO ( Manni *et al.*, 2021) was used to assess gene-set completeness. DIAMOND BLASTp searches of extracted BUSCO proteins and DIAMOND BLASTx searches of assembly sequences against UniProt Reference Proteomes ( Buchfink *et al.*, 2021), followed by BLASTn searches of sequences without DIAMOND BLASTx hits against the NCBI nt database ( Altschul *et al.*, 1990), supported taxonomic assignment. Coverage, sequence composition, taxonomic assignment and BUSCO results were integrated into a BlobDir for interactive inspection and visualisation. The snail and blob plots were rendered locally from this hosted BlobDir using BlobTk v0.8.3. The scaffold-length radial scale function for the snail plot was set to linear ( Challis & Blaxter, 2026). The blob plot used circular markers with area proportional to scaffold length ( Challis *et al.*, 2020).

## Genome sequence report

### Sequence data

The genome of a specimen of *R. nigrum* was sequenced using Pacific Biosciences single-molecule HiFi long reads, generating 75.12 Gb (gigabases) from 6.61 million reads. These reads were used to assemble the genome. GenomeScope analysis using a *p* = 2 model gave a *k*-mer-based haploid genome size estimate of 902.54 Mb, with heterozygosity of 0.87% and repeat content of 57.65%; the full-model fit was 96.17% (Figure 2). Using flow cytometry, the genome size (1C‐value) of the sample was estimated to be 1.1 pg, equivalent to 1,080.00 Mb. These estimates guided expectations for the assembly. The NCBI assembly record reports 81× genome coverage. Hi-C sequencing produced 94.48 Gb from 312.86 million read pairs, which were used to scaffold the haplotype 1 assembly. RNA sequencing data were also generated and are available in public sequence repositories. Table 1 summarises the specimen and sequencing details.

**Figure 2.**
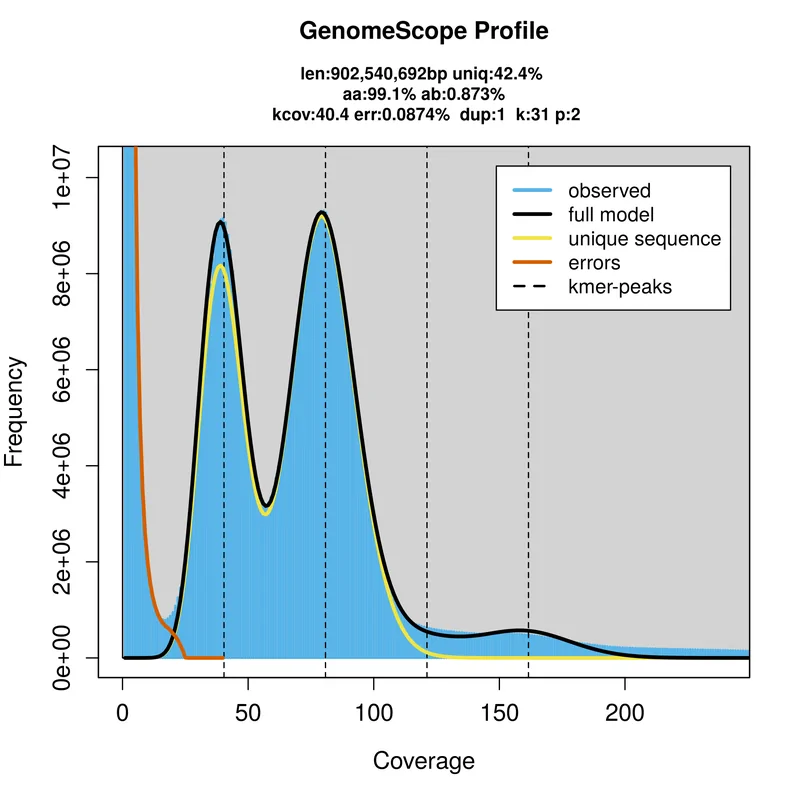
GenomeScope model fitted to the *k*-mer frequency distribution. The plot shows the observed and modelled *k*-mer spectra used to estimate haploid genome size, heterozygosity and repeat content from unassembled sequencing reads.

**Table 1.** Specimen and sequencing data for *R. nigrum* (BioProject PRJEB81059)

**Platform**	**PacBio HiFi**	**Hi-C**	**RNA-seq**
**ToLID**	drRibNigr1	drRibNigr1	drRibNigr1
**Specimen ID**	KDTOL10979	KDTOL10979	KDTOL10979
**BioSample (source individual)**	SAMEA114565234	SAMEA114565234	SAMEA114565234
**BioSample (tissue)**	SAMEA114565302	SAMEA114565302	SAMEA114565302
**Tissue**	leaf	leaf	leaf
**Instrument**	Revio	Illumina NovaSeq X	Illumina NovaSeq X
**Library ID & run accession(s)**	DTOL14819228: ERR13800610	SQPP-60813-N:B2: ERR13802744	ERR14379147
**Read count total**	6.61 million reads	312.86 million read pairs	47.54 million read pairs
**Base count total**	75.12 Gb	94.48 Gb	14.36 Gb

### Assembly statistics

The genome was assembled into two haplotypes using Hi-C phasing. The haplotype 1 assembly was curated to chromosome level, while haplotype 2 was assembled to scaffold level. The haplotype 1 assembly, which is proposed as the reference assembly for this species, has a total length of 894.28 Mb in 509 scaffolds (excluding organelle sequences), with 475 gaps, and its scaffold N50 is 111.81 Mb (Table 2). The scaffold auN for the haplotype 1 assembly is 105 Mb.

**Table 2.** Genome assembly statistics for *R. nigrum*

**Genome assembly**	**Haplotype 1**	**Haplotype 2**
**Assembly name**	drRibNigr1.hap1.2	drRibNigr1.hap2.2
**Assembly accession**	GCA_965278215.2	GCA_965278195.2
**Assembly level**	chromosome	scaffold
**Span (Mb)**	894.28	883.57
**Number of chromosomes**	8	-
**Number of contigs**	984	832
**Contig N50 (Mb)**	3.22	3.25
**Number of scaffolds (excluding organelle sequences)**	509	382
**Scaffold N50 (Mb)**	111.81	111.16
**Scaffold auN (Mb)**	105	103
**Longest scaffold length (Mb)**	127.94	128.39
**Organelles**	Mitochondrial genome: 450.22 kb; Plastid genome: 157.45 kb	-

Most of the haplotype 1 assembly sequence (96.1%) was assigned to 8 chromosome-level scaffolds. These chromosome-level scaffolds, confirmed by Hi-C data, are named according to size (Figure 3; Table 3). During curation we observed a heterozygous inversion on Chromosome 1 ~51.2–62.5 Mbp.

The mitochondrial genome (length 450.22 kb, OZ256409.1) and plastid genome (length 157.45 kb, OZ463007.1) were also assembled. These sequences are included as contigs in the multifasta file of the haplotype 1 genome submission and as standalone records.

**Figure 3.**
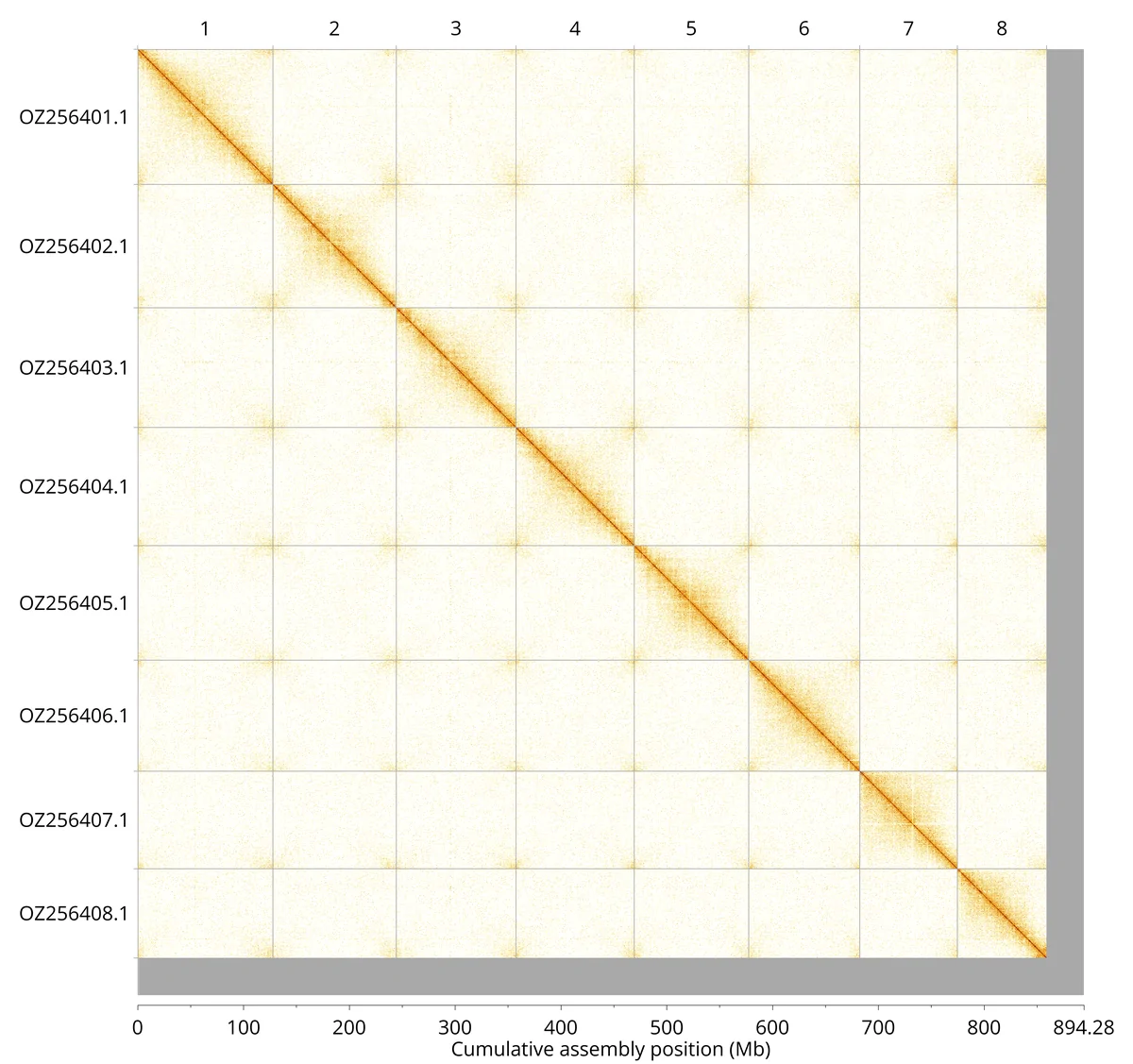
Hi-C contact map of the *R. nigrum* haplotype 1 assembly. Assembled chromosomes are shown in order of size and labelled along the axes, with a megabase scale shown below. The plot was generated using PretextSnapshot.

**Table 3.** Chromosomal pseudomolecules in the haplotype 1 genome assembly of *R. nigrum* drRibNigr1

**INSDC accession**	**Molecule**	**Length (Mb)**	**GC%**
OZ256401.1	1	127.94	37
OZ256402.1	2	116.56	37
OZ256403.1	3	113.11	37
OZ256404.1	4	111.81	37
OZ256405.1	5	108.15	37
OZ256406.1	6	105.02	37
OZ256407.1	7	92.29	37
OZ256408.1	8	84.48	37

### Assembly quality metrics

For the haplotype 1 assembly, the estimated QV is 65.4, and for the haplotype 2 assembly, 64.5. When the two haplotypes are combined, the assembly achieves an estimated QV of 64.9. The *k*-mer completeness is 82.71% for the haplotype 1 assembly, 82.33% for the haplotype 2 assembly, and 99.02% for the combined haplotypes (Figure 4).

BUSCO analysis using the eudicots_odb10 reference set ( *n* = 2,326) identified 98.5% of the expected gene set (single = 93.6%, duplicated = 4.9%) in the haplotype 1 assembly. For the haplotype 2 assembly, BUSCO v.5.7.1 analysis identified 98.3% of the expected gene set (single = 94.0%, duplicated = 4.3%).

**Figure 4.**
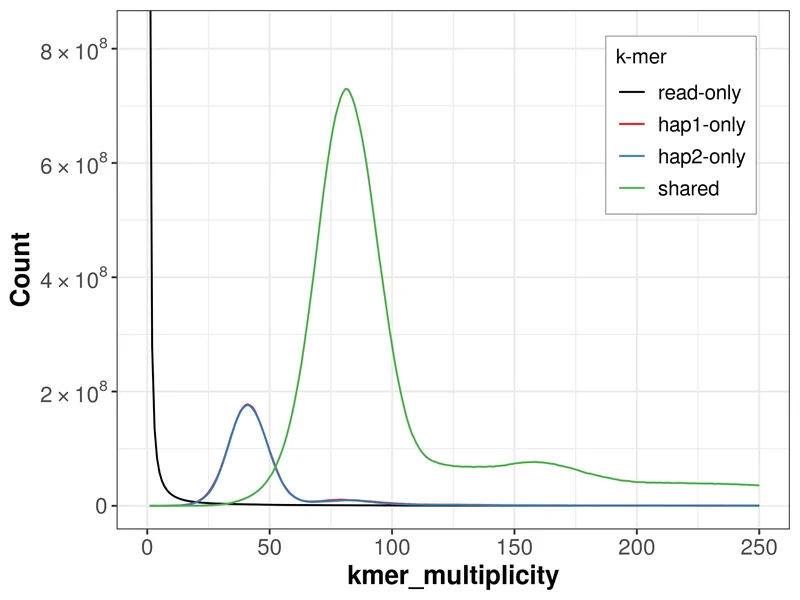
Evaluation of *k*-mer completeness using MerquryFK. This plot illustrates the recovery of *k*‐mers from the original read data in the haplotype 1 assembly, the haplotype 2 assembly and the combined haplotypes. The horizontal axis represents *k*‐mer multiplicity, and the vertical axis shows the number of *k*‐mers. The black curve represents *k*‐mers that appear in the reads but are not assembled, while the remaining curves correspond to *k*‐mers recovered in the assemblies shown.

The snail plot in Figure 5 provides a summary of assembly contiguity, cumulative scaffold count, sequence composition and BUSCO completeness for the haplotype 1 assembly ( Challis & Blaxter, 2026). The relative sizes of the longest scaffold, scaffold N50 and N90, together with the dark-grey scaffold-length distribution, show how much of the assembly is contained in long scaffolds. The outer composition track shows variation in GC, AT and N content, while the central track shows the cumulative scaffold count and the BUSCO inset summarises gene-set completeness.

The blob plot in Figure 6 displays scaffolds from the haplotype 1 assembly by GC proportion and base coverage. Marker area is proportional to scaffold length, and colour indicates taxonomic assignment ( Challis *et al.*, 2020).

**Figure 5.**
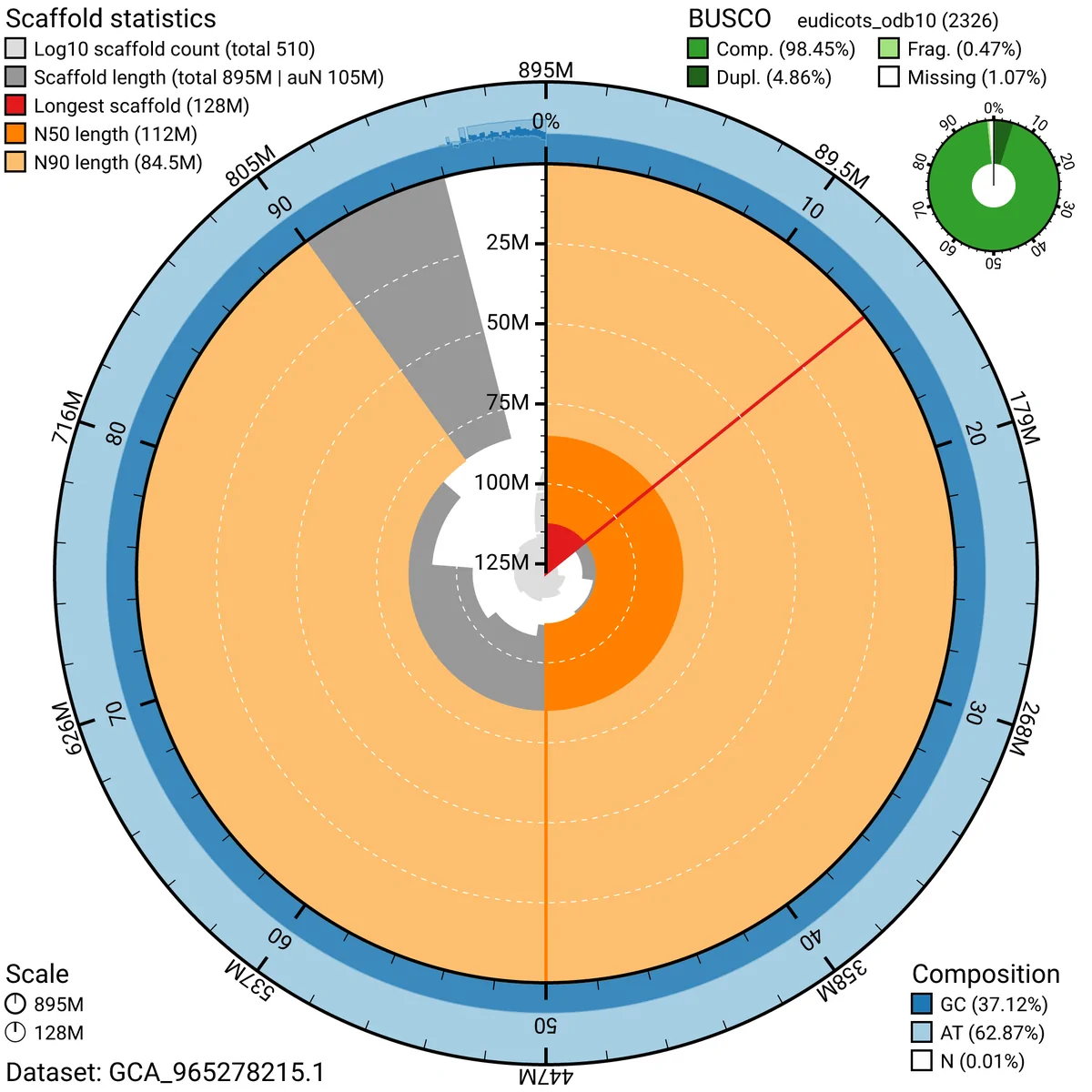
Assembly metrics for drRibNigr1.hap1.2, the haplotype 1 assembly. The BlobToolKit snail plot provides an overview of assembly metrics and BUSCO gene completeness. The circumference represents the length of the haplotype 1 assembly, and the main plot is divided into 1,000 bins around the circumference. The outermost blue tracks display the distribution of GC, AT, and N percentages across the bins. Scaffolds are arranged clockwise from longest to shortest and are depicted in dark grey. Scaffold length is shown on a linear radial scale, scaled to the longest scaffold. Red shading indicates the longest scaffold, and the deeper orange and pale orange shading represent the N50 and N90 lengths. The summary statistics include scaffold auN, the area under the scaffold Nx curve. The light grey spiral at the centre shows the cumulative scaffold count on a logarithmic scale. A summary of complete, fragmented, duplicated, and missing BUSCO genes in the eudicots_odb10 set is presented at the top right. The assembly can be explored interactively in the BlobToolKit viewer.

**Figure 6.**
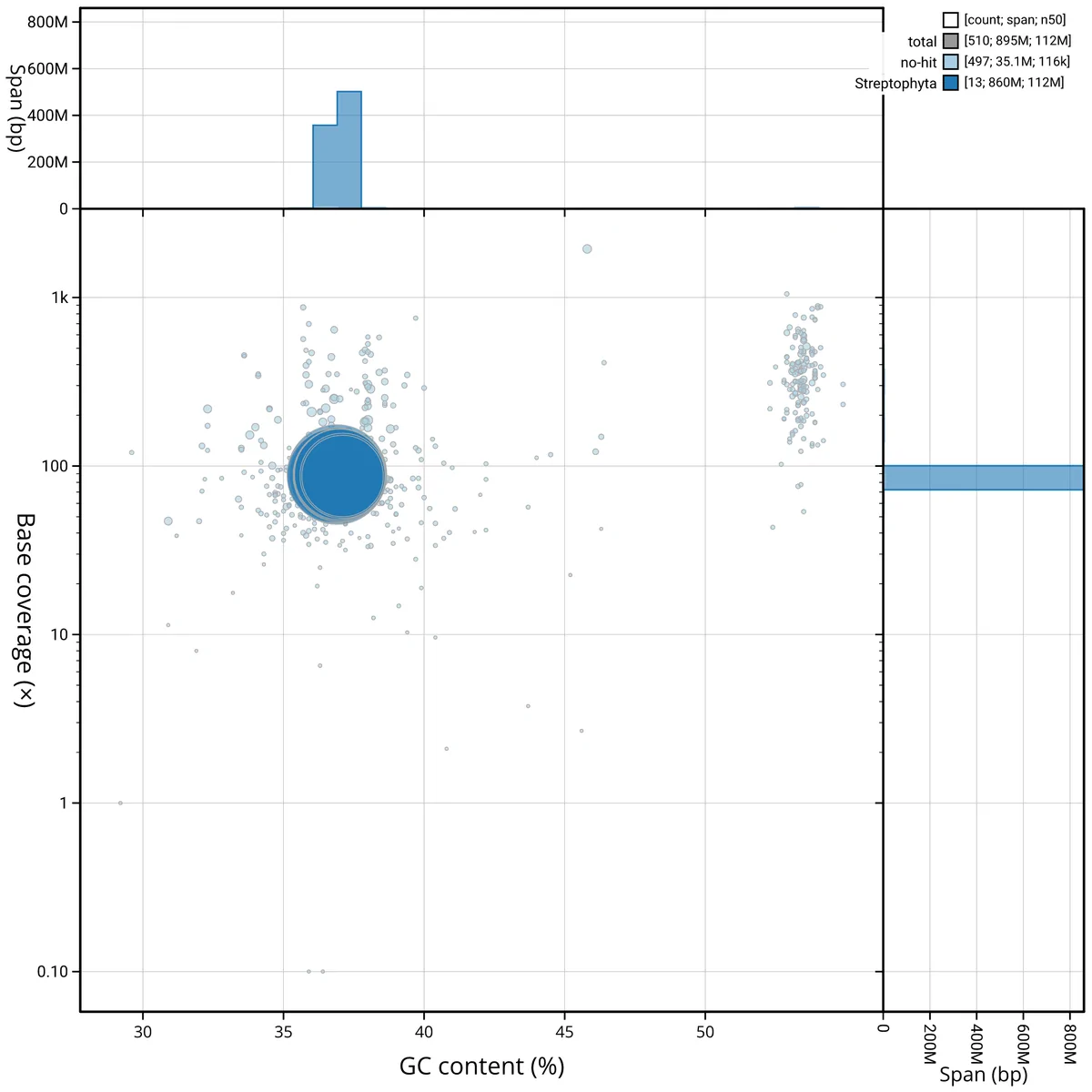
BlobToolKit blob plot for drRibNigr1.hap1.2, the haplotype 1 assembly. The plot shows base coverage (vertical axis) and GC content (horizontal axis). Circles represent scaffolds; marker area is proportional to scaffold length, and colour indicates taxonomic assignment. The histograms along the axes display the total length of sequences distributed across different levels of coverage and GC content. The assembly can be explored interactively in the BlobToolKit viewer.

Table 4 presents assembly quality metrics alongside the Earth BioGenome Project (EBP) benchmarks described by Howe *et al.*. (2026). The three-part EBP metric calculated for the haplotype 1 assembly is **6.C.Q65**.

**Table 4.** Earth BioGenome Project summary metrics for the *R. nigrum* assembly

**Measure**	**Value**	***Benchmark***
EBP summary (haplotype 1)	6.C.Q65	*6.C.Q40*
Contig N50 length	3.22 Mb	*> 1 Mb*
Scaffold N50 length	111.81 Mb	*= chromosome N50*
Consensus quality (QV)	Haplotype 1: 65.4; haplotype 2: 64.5; combined: 64.9	*≥ 40*
*k*-mer completeness	Haplotype 1: 82.71%; Haplotype 2: 82.33%; combined: 99.02%	*> 90% (combined assembly)*
BUSCO	Haplotype 1: C:98.5% [S:93.6%, D:4.9%], F:0.5%, M:1.1%, n:2,326; Haplotype 2: C:98.3% [S:94.0%, D:4.3%], F:0.4%, M:1.2%, n:2,326	*C > 90%*
Percentage of assembly assigned to chromosomes	96.10%	*≥ 90%*

**Notes:** The EBP summary uses log10(Contig N50); chromosome-level (C) or log10(Scaffold N50); Q (Merqury QV). For *k*-mer completeness, the benchmark is assessed using the combined assembly value. BUSCO v5.7.1 using the eudicots_odb10 lineage dataset: C=complete; S=single-copy; D=duplicated; F=fragmented; M=missing; n=orthologues. The BUSCO benchmark for plants uses total completeness (C) to accommodate biological duplication, as an adaptation of the EBP single-copy benchmark.

### Data availability

European Nucleotide Archive: Ribes nigrum. Accession number PRJEB81059; https://identifiers.org/ena.embl/PRJEB81059. The genome sequence is released openly for reuse. The *R. nigrum* genome sequencing initiative is part of the Darwin Tree of Life Project ( PRJEB40665) and the Sanger Institute Tree of Life Programme ( PRJEB43745). All raw sequence data and the haplotype assemblies have been deposited in INSDC databases. The genome will be annotated using available RNA-Seq data and presented through the Ensembl pipeline at the European Bioinformatics Institute. Raw data and assembly accession identifiers are reported in Table 1 and Table 2.

Pipelines used for genome assembly at the WSI Tree of Life are available at https://pipelines.tol.sanger.ac.uk/pipelines. Table 5 lists software versions used in this study.

**Table 5.** Software versions and sources

**Software**	**Version**	**Source**
BLAST	2.14.0	https://ftp.ncbi.nlm.nih.gov/blast/executables/blast+/
BlobToolKit	4.4.5	https://github.com/genomehubs/blobtoolkit
BlobTk (hosted BlobDir)	0.5.1	https://github.com/genomehubs/blobtk
BlobTk (snail and blob plot renderer)	0.8.3	https://github.com/genomehubs/blobtk
BUSCO	5.7.1; 6.1.0	https://gitlab.com/ezlab/busco
bwa-mem2	2.2.1	https://github.com/bwa-mem2/bwa-mem2
DIAMOND	2.1.8	https://github.com/bbuchfink/diamond
fasta_windows	0.2.4	https://github.com/tolkit/fasta_windows
FastK	1.2	https://github.com/thegenemyers/FASTK
GenomeScope	2.1.0	https://github.com/tbenavi1/genomescope2.0
Gfastats	1.3.6	https://github.com/vgl-hub/gfastats
Hifiasm	0.19.8-r603	https://github.com/chhylp123/hifiasm
HiGlass	1.13.4	https://github.com/higlass/higlass
MerquryFK	1.1.0-c1	https://github.com/thegenemyers/MERQURY.FK
Minimap2	2.28-r1209	https://github.com/lh3/minimap2
Oatk	1.0	https://github.com/c-zhou/oatk
MultiQC	1.14; 1.17 and 1.18	https://github.com/MultiQC/MultiQC
Nextflow	24.10.4	https://github.com/nextflow-io/nextflow
PretextSnapshot	0.0.6	https://github.com/sanger-tol/PretextSnapshot
PretextView	1.0.3	https://github.com/sanger-tol/PretextView
samtools	1.21	https://github.com/samtools/samtools
sanger-tol/ascc	0.1.0	https://github.com/sanger-tol/ascc
sanger-tol/blobtoolkit	v0.7.1	https://github.com/sanger-tol/blobtoolkit
sanger-tol/curationpretext	1.4.2	https://github.com/sanger-tol/curationpretext
Seqtk	1.4-r122	https://github.com/lh3/seqtk
Singularity	3.9.0	https://github.com/sylabs/singularity
TreeVal	1.1.1	https://github.com/sanger-tol/treeval
YaHS	1.2a.2	https://github.com/c-zhou/yahs

### Author information

- Members of the Royal Botanic Gardens Kew Genome Acquisition Lab
- Members of the Plant Genome Sizing Collective
- Members of the Darwin Tree of Life Barcoding collective
- Members of the Wellcome Sanger Institute Tree of Life Management, Samples and Laboratory team
- Members of Wellcome Sanger Institute Scientific Operations – Sequencing Operations
- Members of the Wellcome Sanger Institute Tree of Life Core Informatics team
- Members of the Tree of Life Core Informatics collective
- Members of the Darwin Tree of Life Consortium

### Wellcome Sanger Institute – Legal and Governance

The materials that have contributed to this genome note have been supplied by a Darwin Tree of Life Partner. The submission of materials by a Darwin Tree of Life Partner is subject to the **‘Darwin Tree of Life Project Sampling Code of Practice’**, which can be found in full on the Darwin Tree of Life website. By agreeing with and signing up to the Sampling Code of Practice, the Darwin Tree of Life Partner agrees they will meet the legal and ethical requirements and standards set out within this document in respect of all samples acquired for, and supplied to, the Darwin Tree of Life Project. Further, the Wellcome Sanger Institute employs a process whereby due diligence is carried out proportionate to the nature of the materials themselves, and the circumstances under which they have been/are to be collected and provided for use. The purpose of this is to address and mitigate any potential legal and/or ethical implications of receipt and use of the materials as part of the research project, and to ensure that in doing so we align with best practice wherever possible. The overarching areas of consideration are:

- Ethical review of provenance and sourcing of the material
- Legality of collection, transfer and use (national and international)

Each transfer of samples is further undertaken according to a Research Collaboration Agreement or Material Transfer Agreement entered into by the Darwin Tree of Life Partner, Genome Research Limited (operating as the Wellcome Sanger Institute), and in some circumstances, other Darwin Tree of Life collaborators.
