# Supplementary material for: The genome sequence of black currant, Ribes nigrum L. (Saxifragales: Grossulariaceae)
Source: Wellcome Open Res. 2026 Oct 13;11:871. [Version 1] doi: 10.12688/wellcomeopenres.28122.1 (PMC13722861; doi:10.12688/wellcomeopenres.28122.1)
Supplement: Peer review history

# Reviewer report: Reviewer response for version 1

**DOI:** 10.21956/wellcomeopenres.30941.r174053

**Published:** 2026-10-30

**Authors:** Junting Feng

**Affiliations:** 1 Institute of Tropical Bioscience and Biotechnology, Chinese Academy of Tropical Agricultural Sciences, Haikou, Hainan, China

Black currant has important medicinal and economic value and is also an important component of the Darwin Tree of Life Project. This manuscript provides a detailed description of the methods for genome contig assembly, scaffolding, and manual correction, and includes a comprehensive quality assessment of both genome assembly and annotation. But, the Data Availability section does not include the raw sequencing data or genome annotation files.

Are the datasets clearly presented in a usable and accessible format, and the assembly and annotation available in an appropriate subject-specific repository?

Partly

Are sufficient details of the sequencing and extraction, software used, and materials provided to allow replication by others?

Yes

Are the rationale for sequencing the genome and the species significance clearly described?

Yes

Are the protocols appropriate and is the work technically sound?

Yes

Reviewer Expertise:

Horticulture, Genomics

I confirm that I have read this submission and believe that I have an appropriate level of expertise to confirm that it is of an acceptable scientific standard.

# Reviewer report: Reviewer response for version 1

**Published:** 2026-10-13

**Authors:** Automated Benchmarking

**Affiliations:** 1 Wellcome Sanger Institute, Hinxton, UK

As this article is a Genome Note, Automated Benchmarking has been used to determine whether the reported assembly passes four defined checks established by the Earth BioGenome Project: the raw and assembled data are publicly available, the Earth BioGenome Project three-part metric is met, the k-mer completeness exceeds 90%, and the BUSCO score passes the stated threshold. For Ribes nigrum (drRibNigr1), all four checks passed. Data available: pass. ENA study PRJEB81059, raw-read accessions ERR13800610, ERR13802744, and ERR14379147, and assembly accessions GCA_965278215.2 and GCA_965278195.2 are public. Earth BioGenome Project three-part metric: pass. Haplotype 1 is reported as 6.C.Q65, meeting the 6.C.Q40 reference standard. k-mer completeness: pass. The combined haplotype assemblies have 99.02% k-mer completeness, exceeding the Earth BioGenome Project target of 90%. BUSCO: pass. Haplotype 1, which is proposed as the reference assembly for this species, has 98.5% complete BUSCOs (93.6% single-copy and 4.9% duplicated). BUSCO software version: 5.7.1; lineage dataset: eudicots_odb10. It meets the plant-specific target of greater than 90% complete BUSCOs. This decision uses values reported in the Genome Note and public accession records.

Reviewer Expertise:

NA

I confirm that I have read this submission and believe that I have an appropriate level of expertise to confirm that it is of an acceptable scientific standard.
